# Optimizing human-robot handovers: the impact of adaptive transport methods

**DOI:** 10.3389/frobt.2023.1155143

**Published:** 2023-07-13

**Authors:** Marco Käppler, Ilshat Mamaev, Hosam Alagi, Thorsten Stein, Barbara Deml

**Affiliations:** ^1^ Institute of Human and Industrial Engineering (ifab), Karlsruhe Institute of Technology (KIT), Karlsruhe, Germany; ^2^ Intelligent Process Automation and Robotics Lab (IAR–IPR), Institute for Anthropomatics and Robotics, Karlsruhe Institute of Technology (KIT), Karlsruhe, Germany; ^3^ BioMotion Center, Institute of Sports and Sports Science (IfSS), Karlsruhe Institute of Technology, Karlsruhe, Germany

**Keywords:** human-robot handover, adaptive transport methods, predictability, motor learning, physical handover time, early handover intervention

## Abstract

Humans are increasingly coming into direct physical contact with robots in the context of object handovers. The technical development of robots is progressing so that handovers can be better adapted to humans. An important criterion for successful handovers between robots and humans is the predictability of the robot for the human. The better humans can anticipate the robot’s actions, the better they can adapt to them and thus achieve smoother handovers. In the context of this work, it was investigated whether a highly adaptive transport method of the object, adapted to the human hand, leads to better handovers than a non-adaptive transport method with a predefined target position. To ensure robust handovers at high repetition rates, a Franka Panda robotic arm with a gripper equipped with an Intel RealSense camera and capacitive proximity sensors in the gripper was used. To investigate the handover behavior, a study was conducted with *n* = 40 subjects, each performing 40 handovers in four consecutive runs. The dependent variables examined are physical handover time, early handover intervention before the robot reaches its target position, and subjects’ subjective ratings. The adaptive transport method does not result in significantly higher mean physical handover times than the non-adaptive transport method. The non-adaptive transport method does not lead to a significantly earlier handover intervention in the course of the runs than the adaptive transport method. Trust in the robot and the perception of safety are rated significantly lower for the adaptive transport method than for the non-adaptive transport method. The physical handover time decreases significantly for both transport methods within the first two runs. For both transport methods, the percentage of handovers with a physical handover time between 0.1 and 0.2 s increases sharply, while the percentage of handovers with a physical handover time of >0.5 s decreases sharply. The results can be explained by theories of motor learning. From the experience of this study, an increased understanding of motor learning and adaptation in the context of human-robot interaction can be of great benefit for further technical development in robotics and for the industrial use of robots.

## 1 Introduction

As the development of robots continues, there is growing scientific interest in the direct physical interaction of robots with humans (Goodrich and Schultz, 2007). In the field of assembly, for example, a tool can be handed to an assembler without the assembler having to take his or her eyes off the task at hand. Formally, an object handover can be defined as the joint action of a giver and a receiver who deliberately coordinate their actions to transfer an object. This frequent collaborative action between humans requires a complex interplay of prediction, perception, action, learning, and adaptation by both parties. Implementing a robot-human handover that is as efficient and fluid as the exchange between humans is considered an open challenge for the robotics research community (Ortenzi et al., 2021). Technological developments are influencing novel ways for robots to function, such as motion profiling, velocity profiling (Shibata et al., 1995; Huber et al., 2008), grip force control (Chan et al., 2013), camera-based hand tracking (Zhang et al., 2020) and sensing (Mamaev et al., 2021) combined with machine learning. The goal of all these developments is to achieve the best possible object handover between robots and humans. Successful handovers between humans represent a benchmark to be achieved in robotics in terms of speed, fluidity, and acceptance (Glasauer et al., 2010; Strabala et al., 2013; Ortenzi et al., 2021).

The handover of an object is characterized by complex cognitive and physical processes that allow humans action to be coordinated in interaction with other humans (Ortenzi et al., 2021). Humans use speech, gaze behavior, body movements, and hand and arm postures to communicate the intention to hand over *what* as well as *when* and *where* it will be done (Strabala et al., 2013; Ortenzi et al., 2021). The *what* component refers to what kind of action the interaction partner will perform and what his or her intentions are. The *when* component, on the other hand, is relevant for temporal coordination. Information about when the other person will perform an action and how long it will take contributes to fluid and accurate timing. For spatial coordination in a shared action space, it is important to be able to make predictions about *where* the other person, as well as the objects they are handling, will be in a future position (Sebanz and Knoblich, 2009). According to Castiello (2003), successful collaboration requires understanding the actions and intentions of the people involved. In addition, it is necessary to take into account the actions of the interaction partners in their actions to ensure joint coordination (Glasauer et al., 2010). In the process, the partners agree on various framework conditions and aspects of implementation. These can be agreed upon explicitly through verbal communication or indirectly and adaptively in the course of the interaction through nonverbal communication (Huber et al., 2008). Communicative signals allow us to make predictions about the actions of others. Prediction facilitates the coordination between the interaction partners (Strabala et al., 2012; Strabala et al., 2013; Belhassein et al., 2022). If humans were merely reactive, they would not be able to achieve the fluidity and speed of coordination that is required in many collaborative activities (Sebanz and Knoblich, 2009).

At the present time, several theories and models exist that explain the mutual adaptation of joint actions (Sebanz et al., 2006; Vesper et al., 2010; Knoblich et al., 2011). At the core of adaptation to a human partner is the predictability of the partner’s actions. In contrast to self-generated actions, such as a simple object manipulation, human-to-human object handovers represent only semi-predictable tasks, as there is no precise knowledge for humans about the partner’s future movement behavior (Brand et al., 2022). The sensory effects of one’s own actions can be well anticipated in most cases because the underlying processes of motion control are available for this prediction (Wolpert and Ghahramani, 2000) or even represented as such (Prinz, 1997; Hommel, 2009; 2019). People have a basic internal model of another person’s body and can estimate its current state from visual information (Mason and Mackenzie, 2005), yet the predictability of movement is limited because no information about the other person’s motor commands is available (Blakemore and Decety, 2001). Because other people’s action control processes are not accessible, the challenge is to predict others’ actions and match one’s own actions to them, even though humans themselves do not have direct access to other people’s action control (Vesper, 2020). This means that predictions must be based on other people’s observable movements (Wolpert et al., 2003; Wilson and Knoblich, 2005; Vesper, 2020), which may make them less accurate than anticipating one’s own sensory action effects. The extensive practice that people undergo over the course of their lives may allow them to predict the behavior of their interaction partner based on knowledge of their situation (Aglioti et al., 2008; Ikegami and Ganesh, 2017). Brand et al. (2022) hypothesize that motor control during object handovers involves a sophisticated and commonly established etiquette of feedforward and feedback mechanisms that is implicitly shared within the dyad of two interacting individuals. If this is possible, humans could anticipatorily adjust their own control based on the current state of their partner.

In robot-human object handovers, the human partner has no internal models available to predict the robot’s behavior. Accordingly, the human partner must plan and adapt its behavior based on the robot’s action monitoring to ensure fast and reliable object handovers. Depending on how many degrees of freedom a robot has, it is difficult to nearly impossible for users to estimate the location of the handover based on the robot’s potential kinematics. In motor neuroscience the process of motor adaptation is described as a process in which the motor system responds to changes in the body and/or the environment to return to a previous level of performance under these new conditions (Krakauer and Mazzoni, 2011). In order to adapt to the robot, the human must make a prediction based on certain parameters of the robot. The predictability of the trajectory and the robot’s target position can be considered as appropriate parameters for handovers. For handovers that are re-planned at each handover to adapt to the position of the human partner’s hand (Ortenzi et al., 2021), this prediction will be more difficult than for handovers that always navigate to the same handover location on the same trajectory. According to Sebanz and Knoblich. (2009), the physical handover must be much more reactive due to the poorer prediction of the movement trajectory, which has the consequence that humans cannot realize such a fluid and fast coordination of actions.

**FIGURE 1:**
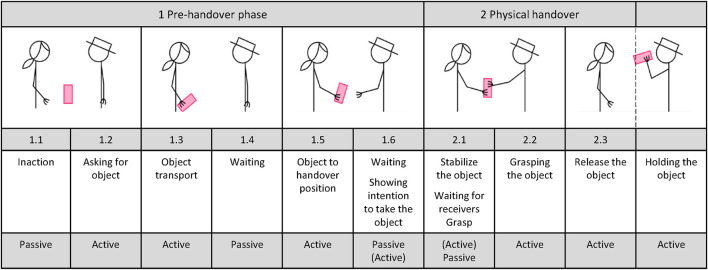
Phasing of human-human object handovers with directed attention of the receiver to the handover object with the addition of the attributes “active” and “passive” (mod. according to Ortenzi et al., 2021).

Ortenzi et al. (2021), in line with other authors (Mason and Mackenzie, 2005), divide handovers into a pre-handover phase and a physical handover. The pre-handover phase includes explicit and implicit communication between the partners involved, as well as the grasping and transporting of the object by the giver. The physical handover begins with the first contact of the receiver’s hand with the object. This phase is completed when the giver removes his hand from the object and the object is completely in the hand of the receiver. To better understand human behavior in an object handover, we add another aspect to the phase classification presented by Ortenzi et al. (2021). This involves assigning an active and a passive attribute to the giver and receiver for each action in the respective phase (Figure 1). Using this description, we want to illustrate that the receiver behaves actively at two key points of the object handover. First, by actively expressing the desire to receive a particular object (1.2), and second, by actively reaching for the object to initiate the physical interaction (2.2). Based on these explanations, we assume that a highly adaptive handover that deliberately moves the handover object toward the receiver’s hand forces the receiver into a passive role of waiting instead of an active role of grasping the object. This circumstance may result in the final decision to take the object being taken away from the initiator of the actual handover action, which may lead to poorer handover performance in terms of speed, fluency, and subjective perception.

Currently, it is unclear what the consequences are when the robot tries to adapt to the human while the human tries to adapt to the robot. The main goal of this study is to better understand whether handovers adapted to the human hand lead to higher overall handover performance than handovers with a fixed robot target position, or *vice versa*. In the case of object handovers between robots and humans, it is important to understand that the overall handover performance is a result of the interaction between robot and human. Based on the assumption of worse predictability of the trajectory and the target position of the adaptive transport method, it is first expected that the adaptive transport method leads to higher physical handover times than the non-adaptive transport method. Second, the non-adaptive transport method is expected to result in earlier handover interventions before the robot reaches its target position than the adaptive transport method. Third, the adaptive transport method is expected to result in lower subjective ratings of the handover than the non-adaptive transport method. Furthermore, there are no studies to date that have performed robot-human handovers over multiple runs with the same conditions and high numbers of repetitions. We assume that most studies in the field of robot-human or human-robot handovers have been conducted with too few repetitions and too few runs to produce meaningful results for the evaluation of human behavior or even for the evaluation of robotic systems. Accordingly, it is assumed that the subjects’ adaptation to the handover task and the robot over time will be reflected in all dependent variables. We assume that the physical handover time will decrease over the course of the runs, that the handover will be intervened earlier in the course of the runs, and that the subjective ratings will either improve or deteriorate.

For this purpose, a study with two experimental groups was conducted. One experimental group performed handovers with the adaptive transport method, the other experimental group performed handovers with the non-adaptive transport method. To evaluate the handover performance, the physical handover time, the early handover intervention before the robot reaches its target position, and the subjective perception of the subjects are compared.

## 2 Materials and methods

### 2.1 Experimental setup

The technical setup of the experiment consists of a table on which a collaborative robot (model Panda by Franka Emika) is mounted. The robot has seven degrees of freedom and a payload of 3 kg. Both a 2-jaw gripper and an Intel RealSense RGB D camera are mounted on the end effector of the robot. Capacitive proximity sensors are integrated in the gripper jaws.

In the experiment, a velocity controller was implemented using the Franka velocity Cartesian interface within the ROS framework. The controller expects a 6D pose vector as input. A velocity vector is calculated based on the remaining distance to the target and the robot’s current velocity, while considering the soft robot limits: a maximum velocity of 0.7 m/s, a maximum acceleration of 6 m/s^2^, and a maximum jerk of 600 m/s³. The control loop operates at 1 kHz, making the velocity controller feel natural and smooth for users. In the non-adaptive control policy, the target position for the robot is predefined, and the robot’s trajectory is maintained for all handovers in the non-adaptive mode. For the adaptive control policy, the 6D pose of the receiver’s hand is tracked and used to update the position of the human hand as the robot’s target. The update frequency is 30 Hz, which means no noticeable delay is visible to the naked eye.

For stopping the robot there are two different options available: hard stop and soft stop. The hard stop, on one hand, ignores any limits and results in an abrupt halt. This function is only used for the emergency stop. The soft stop on the other hand gradually reduces the robot’s velocity to zero while adhering to the robot’s limits. This method was used by default for all handovers. The implemented controller also takes into account the robot’s limited working space, disregarding target positions outside of it. This helps prevent collisions with the table and exceeding the physical limits of the robot.

**FIGURE 2:**
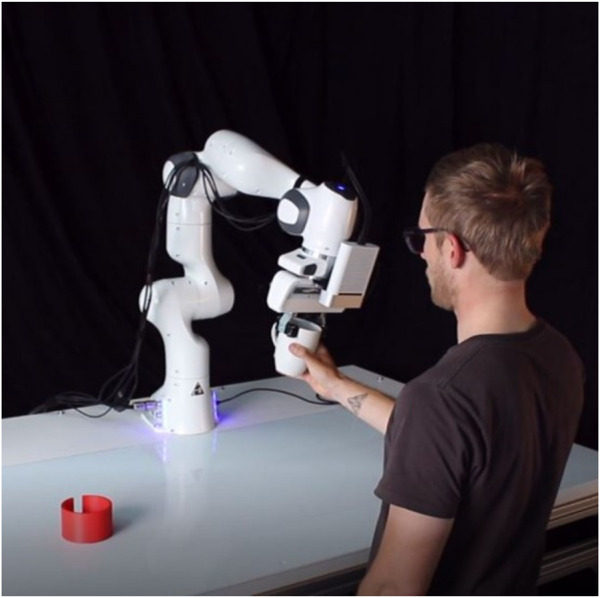
Technical setup of the handover experiment with Panda robot arm and gripper by Franka Emika.

A ceramic cup is used as the handover object. The cup is initially placed in the red container on the left side of the table. The subjects stand in a predefined position at the front of the table (Figure 2). The position was chosen so that the handover space for both the 5th female percentile and the 95th male percentile lies between the preferred and the maximum working range of the right arm (DIN EN ISO 14738, 2008). The subjects are in their starting position outside the reachable space of the robot, they can enter the collaborative workspace by extending their arm. The physical handover of the cup takes place in the area of the right third of the table in the preferred right workspace of the subjects.

### 2.2 Procedure of an object handover

**FIGURE 3:**
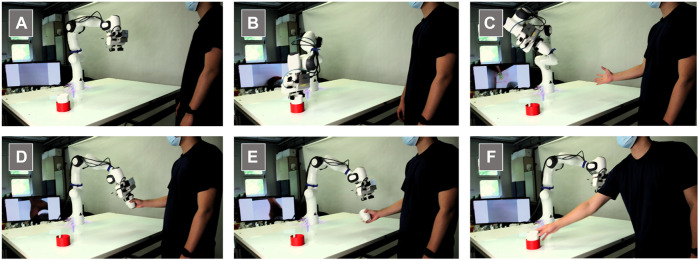
Sequence of a handover: **(A)** initial position of the robot, **(B)** robot grasps the cup, **(C)** subject gives the signal to start handover, **(D)** subject grasps cup, **(E)** robot releases cup, **(F)** subject returns cup.

At the beginning of the handover experiment, the gripper of the Franka Panda is located above the center of the table (Figure 3). This position is called the start position. From this starting position, the cup in the red container is approached. The next step is to grasp the cup. To do this, the robot first moves to a position above the cup, opens the gripper, and moves down to the cup until the gripper’s jaws are at the height of the cup. When this position is reached, the jaws close and the cup is securely gripped. With the cup gripped, the robot then moves to the overview position. This position is chosen so that the subject’s hand can be tracked by the camera. Google MediaPipe Hands, an approach from Google Research’s MediaPipe framework that uses machine learning to ensure fast and robust hand and finger tracking, is used to detect the subject’s hand (Zhang et al., 2020).

In this study, two different transport methods are used for the object handovers. The two transport methods are called the adaptive transport method and the non-adaptive transport method. In the adaptive transport method, the handover space is first scanned for a hand. This allows the subjects to control if and when the robot starts to move. As soon as a hand is detected, the robot arm starts to move, aimingat the hand as its target point. Based on the camera data, the robot can adjust its position to a changed hand position up to 30 cm before reaching the hand. Once the distance to the hand is less than 30 cm, the position cannot be adjusted. Based on the last image data of the hand, an end position is calculated and approached.

In the non-adaptive transport method, the handover space is also scanned for a hand. In this case, hand tracking is used only as a start signal to start the handover process. Once the hand is detected, the robot moves to a previously defined target position. The target position and the traversed path are identical for each handover using the non-adaptive transport method. The target position was chosen to be between the preferred and maximum working range of the subjects’ right arm from an ergonomic point of view. The robot moved at an average speed of 0.3 m/s for all movements.

Capacitive proximity sensors (Alagi et al., 2016) are used to detect the grasp. These sensors are integrated into the gripper jaws of the robot. To detect a grasp, a threshold value was defined that must be exceeded for the gripper to release the cup (Mamaev et al., 2021). Simultaneously with the opening of the gripper, the robot moves up 10 cm to give the subjects more space to grasp the cup. For both transport methods, the subjects can wait for the robot to reach its target position and then grasp the cup, or they can grasp the cup while the robot is still moving toward its target position. After successfully grasping the cup, the subjects place it back into the red container. At the same time, after releasing the cup, the robot moves back to the red container to pick up the cup again. The next handover starts again with the robot grasping the cup.

### 2.3 Sample

Forty students and employees of the Karlsruhe Institute of Technology (KIT) participated in the study (age: 26.33 ± 5.21 years; 25 males). Seven subjects had previous experience with robots and were evenly distributed between the two experimental groups. All subjects were right-handed and performed the experiment with their right hand. All subjects voluntarily participated in the study and were fully informed about the study procedure, their rights, and the anonymity of the data. All subjects signed an informed consent form. There was no compensation for participation in the study.

### 2.4 Experimental procedure

Prior to the study, the subjects were assigned to experimental group *adaptive* or *non-adaptive* in a parallelized manner. The experimenter demonstrated two object handovers to all subjects, each using a transport method appropriate for the experimental group, and explained the handover task. The technical operation of the robot setup was not explained to the subjects. The subjects did not perform a test trial and started directly with the first run of handovers.

**FIGURE 4:**
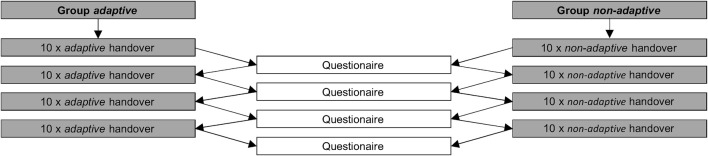
Study design.

Figure 4 provides an overview of the study design. For the subjects, a run consisted of ten consecutive handovers. After the ten handovers, the subjects went to a separate computer and answered five questions about their subjective perception. The questions of the questionnaire could be answered on a 5-point Likert scale and were as follows.

• *Did you perceive the handover as fluid?*
• *Could you easily take the object from the robot?*
• *Do you trust the robot to do the right thing?*
• *Did you feel safe during the handover?*
• *How satisfied are you with the handover overall?*

After answering the questions, the subjects returned to the robotic table and completed the next run. Both experimental groups performed ten handovers in each of the four runs, using the same transport method. The break between the runs was approximately 1 min. Following this pattern, each subject completed a total of four runs. Thus, each subject completed a total of 40 handovers and answered the five questions a total of four times. The total time per subject was approximately 40 min.

### 2.5 Data processing

To study the handover performance, the influence of the transport method, as an independent variable, on the physical handover time, the early handover intervention, and the subjective perception of the subjects, as a dependent variable will be examined at multiple time points.

The physical handover time is measured by the capacitive proximity sensors of the robot and is defined as the time between the first physical contact of the subject with the handover object and the exceeding of the threshold, which then causes the cup to be released. The physical handover time is expressed in seconds.

For the descriptive presentation of the physical handover time data, the data were first examined for outliers. Values >0.5 s were defined as outliers. Based on the outlier analysis, six classes were formed with a distance of 0.1 s from <0.1 s to >0.5 s. The completed handovers were assigned to the defined classes based on their physical handover times and reported as a percentage of all completed handovers in the respective experimental group. The changes in the percentages of the handovers in the course of the four runs are additionally analyzed and differentiated according to the defined classes.

The early handover intervention is defined as the difference vector between the coordinate point of the robot’s overview position and the coordinate point of the object release triggered by the subjects in three-dimensional space:

O_p_ = Overview point; R_p_ = Release point

$$premature\;handover=\left|\vec{O_{p}}-\vec{R_{p}}\right|=\left(\begin{array}{c} {Op}_{x}\\ {Op}_{y}\\ {Op}_{z} \end{array}\right)-\left(\begin{array}{c} {Rp}_{x}\\ {Rp}_{y}\\ {Rp}_{z} \end{array}\right)$$

The lower the value in cm, the closer the object was grasped to the robot’s overview position, and therefore the earlier the participants intervene in the robot’s transport movement.

To test for significant differences in physical handover time and early handover intervention between groups (adaptive, non-adaptive) and runs (1–4), analyses of variance in the form of two-factor mixed ANOVA are performed. Not all data are normally distributed. However, since ANOVA is considered robust to a violation of the normal distribution when sample sizes are equal, it is considered appropriate for this study (Schmider et al., 2010; Wilcox, 2012; Field et al., 2014). ANOVAs are followed by *post hoc* testing with Bonferroni correction. The significance level is set *a priori* at *p* = 0.05. The effect size is given by eta squared (η^2^). If sphericity was violated, the degrees of freedom were adjusted using the Greenhouse-Geisser correction. In order to investigate the subjective perception of the subjects regarding the handover, the data of the answered questionnaires are used. To quantify the differences between the different transport methods, the data were first processed descriptively and plotted. To statistically validate the results, pairwise comparisons of runs 1–4 were calculated using the Wilcoxon test. Group differences in each run were calculated using the Mann-Whitney U test. The effect size is reported using the biserial rank correlation. The significance level was set *a priori* at*p* = 0.05.

## 3 Results

The results section is divided into three subsections that consider the three performance parameters of the study: physical handover time, early handover intervention, and subjective evaluation. The physical handover time is considered in terms of the differences in the mean values, between the study groups and the completed runs. An overview of the percentages of all completed handovers within the class limits over the course of the runs follows. The early handover intervention is considered based on the differences in the mean values between the study groups and the completed runs. The third subchapter presents the answers to the questions about the subjects’ subjective perceptions.

### 3.1 Influence of the transport method on the physical handover time

#### 3.1.1 Comparison of the mean values of the physical handover time

**FIGURE 5:**
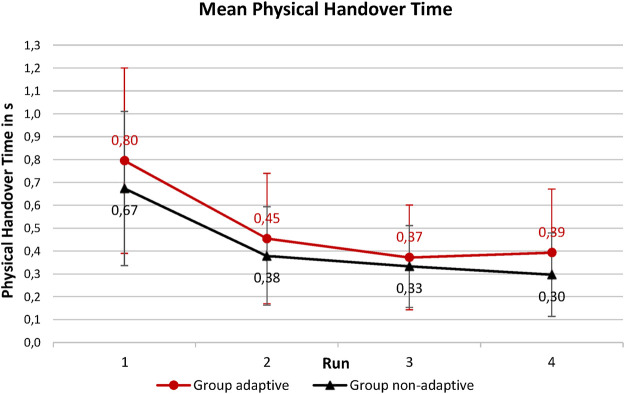
Comparison of the mean physical handover times of the two experimental groups over the course of the four runs.

Figure 5 gives an overview of the progression of the mean physical handover times over the course of the four runs. There is no statistically significant interaction between the runs and the groups [F (3,110) = 0.108, *p* = .959, η^2^ = .001]. There is no significant main effect of groups on physical handover time [F (1, 38) = 1.542, *p* = .222, η^2^ = .012]. Physical interaction time decreases significantly over the course of the runs [F (3,114) = 21.780, *p* < .001, η^2^ = .014]. In group *adaptive*, the physical handover time significantly decreases from 0.80 s (±0.41 s) in the first run to 0.45 s (±0.29 s) in the second run [t (19) = 4.161, p_bonf_ = 0.002, d = 1.076]. In group *non-adaptive*, the physical handover time decreases from 0.67 s (±0.34 s) in the first run to 0.38 s (±0.22 s) in the second run also significantly [t (19) = 3.900, p_bonf_ = 0.006, d = 1.008].

There are no statistically significant differences between experimental groups A (adaptive) and B (non-adaptive), while for both experimental groups, the mean physical handover time decreases significantly from the first to the second run.

#### 3.1.2 Comparison of the percentages of handovers within the class boundaries of the physical handover time

**FIGURE 6:**
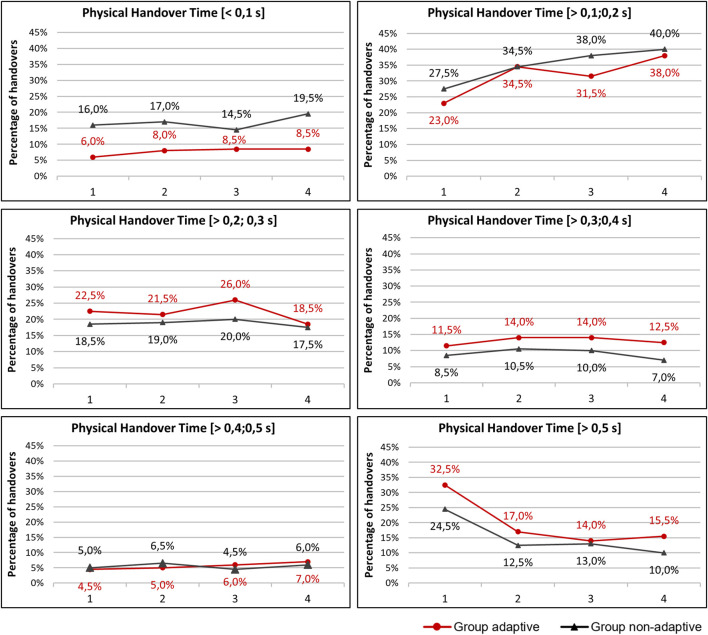
Comparison of the percentages of handovers of both experimental groups within the four runs using the defined class limits of the physical handover time: [< 0.5 s]; [> 0.1; 0.2 s]; [> 0.2; 0.3 s]; [> 0.3; 0.4 s]; [> 0.4; 0.5 s]; [> 0.5 s].

Looking at the mean values, it is clear that the physical handover time decreases over the course of the four runs. At the same time, the mean values show high standard deviations. Six classes were defined to provide a more nuanced view of how the reduction in mean values occurs and in which areas the greatest changes occur. The handovers were assigned to the defined classes separately by runs and study group. Figure 6 gives a descriptive overview of the development of the percentages of handovers within the classes <0.1 s to >0.5 s over the course of the four runs. For each class, the group differences and the differences over the course of the four runs are considered.

In summary, for handovers <0.1 s, the adaptive transport group has lower percentages compared to the non-adaptive transport group. For handovers with handover times between 0.1 s and 0.2 s, there are no major differences in the percentages of the two experimental groups. In both experimental groups, there is an increase in the percentage of handovers between 0.1 s and 0.2 s over the course of the four runs. For handovers between 0.2 s and 0.3 s; 0.3 s and 0.4 s; and >0.5 s, the adaptive transport group has higher percentages compared to the non-adaptive transport group. For handovers >0.5 s, the adaptive transport method results in higher percentages than the non-adaptive transport method. For handovers >0.5 s, both experimental groups experience a decrease in percentages over the course of the first two runs.

The largest percentage increase for both study groups occurred for handovers between 0.1 s and 0.2 s, while the largest percentage decrease for both study groups occurred for handovers >0.5 s.

### 3.2 Influence of the transport method on the early handover intervention in the handover

#### 3.2.1 Comparison of the mean values of the early handover intervention

**FIGURE 7:**
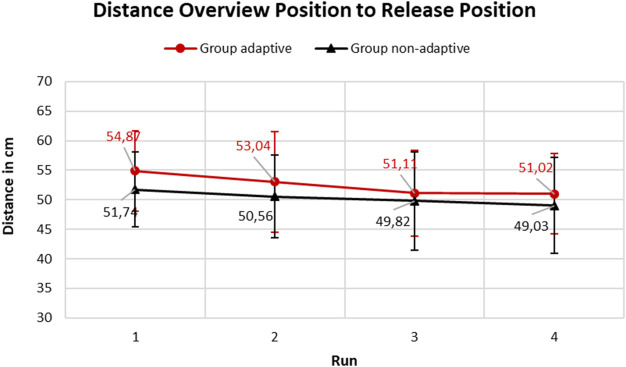
Comparison of the mean values of the early handover intervention, parameterized by the distance between the overview position of the robot and the release position of the object, of the two experimental groups during the four runs.

Figure 7 shows the progression of the mean early intervention scores over the course of the four runs. There is no statistically significant interaction effect between runs one to four and the groups [F (3,114) = 0.696, *p* = 0.556, η^2^ = 0.002]. The effects between the study groups show no significant differences [F (1,38) = 1.010, *p* = 0.321, η^2^ = 0.022]. There is a significant main effect of the runs with earlier handover interventions in the course of the runs [F (3,114) = 10.074, *p* < .001, η^2^ = 0.029]. In the *non-adaptive* group, the handover is not intervened significantly earlier in the course from the first to the fourth run. For group *adaptive*, the handover is intervened significantly earlier in the course from the first to the third [t (19) = 4.036, p_bonf_ = 0.003, d = 0.505], and compared from the first to the fourth run [t (19) = 4.137, p_bonf_ = 0.002, d = 0.517]. Group *adaptive* grasps the handover object 54.87 cm (±6.8 cm) away from the start position in the first run and 51.11 cm (±7.2 cm) away from the start position in the fourth run.

In the experimental group with the *non-adaptive* transport method, the handover object is not grasped significantly earlier from the first to the fourth run. In the *adaptive* transport group on the other hand, the handover object is grasped significantly earlier in the third and fourth run than in the first run.

### 3.3 Influence of the transport method on the subjective evaluation

**FIGURE 8:**
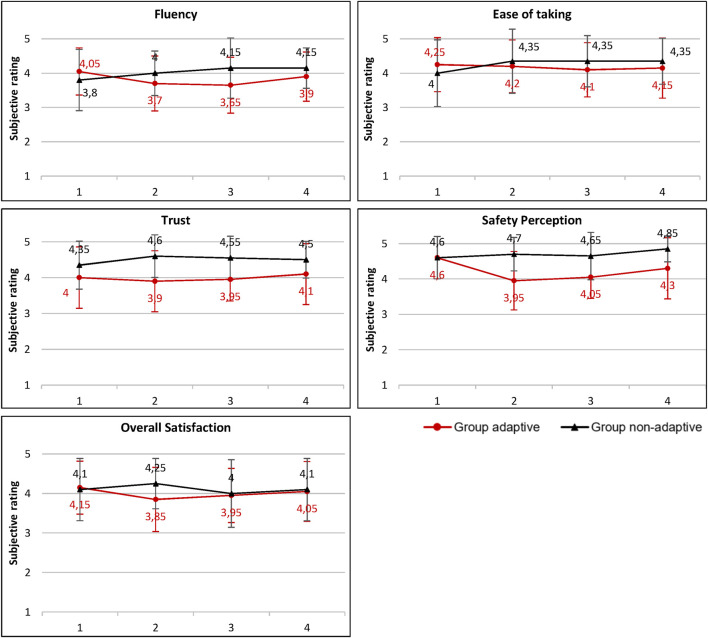
Comparison of the subjective perceptions of the two experimental groups in the four handover runs. Ratings on a 5 point scale from 1: no, not at all; to 5: yes, completely. *Written informed consent was obtained from the [individual(s) AND/OR minor(s)’ legal guardian/next of kin]* for the publication of any potentially identifiable images or data included in this article.

Figure 8 provides an overview of the progression of the subjective ratings over the course of the runs. The evaluation of *trust in the robot* shows no significant differences for group *adaptive* as well as for group *non-adaptive* within the four runs. In the second run, group *adaptive* (3.9 ± 0.85) and group *non-adaptive* (4.6 ± 0.6) show a significant difference in the trust score (W = 106.000, *p* = 0.006, r = −0.470). In the third run, there is also a significant difference (W = 102.500, *p* = 0.004, −0.488) in trust ratings between group *adaptive* (4.0 ± 0.6) and group *non-adaptive* (4.6 ± 0.6).

Group *adaptive*’s rating of *perceived safety* during the handover shows a significant decrease (W = 62.000, *p* = 0.009, r = 0.879) in rating from 4.6 (±0.6) in the first run to 4.0 (±0.83) in the second run. Group *non-adaptive*’s score shows no significant differences across the four runs. In the second run, group *adaptive* (4.0 ± 0.83) and group *non-adaptive* (4.7 ± 0.47) show a significant difference in their ratings of perceived safety (W = 99.000, *p* = 0.003, r = −0.505). In the third run, there is also a significant difference (W = 98.500,*p* = 0.003, r = −0.508) in the evaluation of the *perception of safety* between group *adaptive* (4.1 ± 0.6) and group *non-adaptive* (4.7 ± 0.67). Another significant difference in the *perception of safety* is present in the fourth run (W = 125.500, *p* = 0.015, r = −0.373) between the *adaptive* (4.3 ± 0.86) and the *non-adaptive* group (4.9 ± 0.37).

The transport method shows a significant influence on the *trust in the robot* and the *perception of safety* during the handovers. In contrast, the transport method does not influence the *perceived fluency* of the handover, the *ease of taking*, and the *overall satisfaction* with the handover.

## 4 Discussion

The primary objective of this study was to compare the handover performance of a handover online adapted to the human hand with a handover whose final position was predetermined. For this purpose, a study with two experimental groups was conducted. Group *adaptive* performed 4 × 10 handovers using the adaptive transport method, while group *non-adaptive* performed 4 × 10 handovers using the non-adaptive transport method. Physical handover time, early handover intervention and subjective perception were used as measures to evaluate the handovers.

The adaptive transport method does not result in significantly higher mean physical handover times than the non-adaptive transport method. The non-adaptive transport method does not result in significantly earlier intervention in the handover process than the adaptive transport method. The adaptive transport method results in significantly lower trust and perceived safety ratings than the non-adaptive transport method. The physical handover time decreases significantly within the first two runs for both experimental groups. Early handover intervention increases significantly in the adaptive transport group in the course of the runs. The adaptive transport group showed a significant decrease in the safety perception rating over the course of the runs.

### 4.1 Influence of the transport method on handovers

The adaptive transport method leads to higher mean physical handover times in all runs. However, the differences to the non-adaptive transport method are not significant. The mean values of both transport methods show high standard deviations, which are due to handovers with very high physical handover times. Dividing the handovers into classes of physical handover time gives a more precise insight into the distribution of the handovers. The adaptive transport method results in lower percentages for very fast handovers and higher percentages for slower handovers compared to the non-adaptive transport method. For the early handover intervention, there are no statistical differences between the adaptive and the non-adaptive transport method. The transport method has no significant effect on the subjectively perceived fluency of the handover, the ease of taking the object, and the overall satisfaction with the handover. The same sensor technology was used for the handover of the object in both experimental groups. The function of the gripper accounts for a large part of the grasping and releasing of the object and thus has a large influence on the evaluation of the above-mentioned parameters. Trust in the robot and the perception of safety are rated significantly lower for the adaptive transport method than for the non-adaptive transport method.

In the adaptive transport method, the start signal for the handover is given by the subjects, after which the subjects observe the robot’s movement path, and estimate the target position based on this. It can be assumed that the adaptive transport method is associated with more uncertainties in predicting the robot’s target position, which leads to lower trust and perception of safety. By varying the starting position of the subject’s hand, the robot’s trajectory is adjusted, which can cause the robot’s motion to lose predictability for the subjects. This complicates the predictability of the trajectory and target position compared to the non-adaptive transport method. In the non-adaptive transport method, the subjects get used to the same trajectory and target position more easily, which gives them more freedom in deciding when to intervene and how to hold the hand for the handover. This may explain the higher trust and sense of safety towards the robot. On the other hand, it could also be argued that the robot’s intention is clearer to the subjects with the adaptive transport method because the robot targets the subjects’ hand, whereas the non-adaptive transport method bluntly follows the same trajectory with the same target position. The earlier handover intervention of the adaptive group in the course of the runs could be explained by an increased confidence in the robot. However, this is contradicted by the subjectively significantly lower evaluated confidence and safety perception.

### 4.2 Adaptation to the robot in the course of the runs

A closer look at the studies that have been conducted in the area of human-human, robot-human, and human-robot handovers reveals that they either have small sample sizes or have performed only a small number of handover repetitions, or both in combination (Becchio et al., 2008; Huber et al., 2008; Basili et al., 2009; Dehais et al., 2011; Aleotti et al., 2012; Pan et al., 2018). Depending on the purpose of the study, the small sample size and the small number of repetitions were sufficient to answer the respective research question or to evaluate and advance the respective technical development. However, in agreement with Leichtmann et al. (2022), we see great potential in conducting studies with larger sample sizes and also higher numbers of repetitions, thereby uncovering more details of human behavior in cooperation with robots and making them useful for further research and development.

Through the four runs performed, each with ten handovers, it was shown that both transport methods significantly reduce the mean physical handover time from the first to the second run. The percentage of handovers with a handover time between 0.1 s and 0.2 s in both experimental groups increases sharply over the course of the four runs, regardless of the transport mode. In the class of handovers with handover times >0.5 s, the course of the four runs leads to a strong decrease in the percentages. Based on the physical handover time, two central tendencies of adaptation can be identified in the course of the runs. First, subjects try to reduce handovers with very long interaction times. Second, subjects try to perform handovers with interaction times between 0.1 s and 0.2 s.

From a neuroscientific perspective, visual feedback requires approximately 100 ms (Wolpert et al., 2013) to be processed by the retina and transmitted to the visual cortex. Döhring et al. (2020) propose that tactile sensory feedback about an object can be processed within the first 80–100 ms. Further delays occur in further processing. The combined delay of the sensorimotor loop is approximately 200 ms (Wolpert et al., 2013) until the response to a visual or tactile stimulus. This means that fast movements cannot use sensory feedback. Accordingly, open-loop control must be used to initialize a movement (Wolpert et al., 2013). In most motor systems, motor control is achieved by both processes, feedforward and feedback. Since sensory feedback is not available for the first part of a movement, feedforward processes alone generate the initial motor commands. As the movement progresses, information about the movement becomes available, leading to feedback control to intervene (Wolpert et al., 2013).

Regarding the increase in the percentage of handovers between 0.1 and 0.2 s, one could speculate that the increase in percentages could be explained by a decrease in feedback-based control and an increase in feedforward control. In a previous study of human-to-human handovers, a high number of handovers were performed with an empty plastic cup (Käppler et al., 2021). These handovers were recorded with a marker-based motion capture system and analyzed for their physical handover time. The mean handover time of the empty cup from human to human in this study was ∼0.16 s, which is between 0.1 s and 0.2 s and suggests that there is some kind of optimal speed for the physical handover time here. In this context, it is important to check whether the data from the motion capture recording are comparable to the data generated by the capacitive proximity sensors.

Since the subjects have already performed many object handovers with other people in their lives, they have a certain idea of how fast and smooth an object handover should be. The changes in the listed values can be explained in the context of error-based learning. Through the process of error-based learning, human motor behavior is constantly adapting. When the motor system encounters an error, its assessment of the body and the environment changes, and the next movement is immediately modified to counteract the underlying error (Diedrichsen et al., 2010). If the problems that occur during handovers with the robot are considered perturbations, then the changes in the data can be attributed to motor adaptation. This describes the ability of the motor system to regain its previous performance under changed external or internal conditions (Krakauer and Mazzoni, 2011).

Descriptively, the subjects tend to reduce the distance between the robot’s overview position and the grasping position of the object for both transport methods. For the non-adaptive transport method, this reduction is not significant. For the adaptive transport method, it was shown that the distance between the overview position of the robot and the grasping position of the object is significantly reduced over the course of the runs. A hypothesis by Izawa et al. (2008) assumes that the goal of the nervous system is to maximize performance in a new environment. Internal models are used to search for a better movement plan to minimize the implicit motor effort and maximize the yield of the movement. The property of maximizing the yield of the movement can explain why subjects tend to engage early in the handover. The subjects try to speed up the overall handover process by intervening earlier. At the same time, this approach reduces the amount of information processing required. During the movement of the robot, a continuous observation of the robot arm takes place. Based on the observation of the robot movement, estimations for the best possible intervention location and time are continuously calculated. By intervening earlier in the robot movement, the costs are shifted from an observation effort to an effort for prediction. Whether this actually saves costs remains unclear. In any case, the yield is higher due to the faster executed object handover. Accordingly, the overall balance is positive. In this context, the question arises whether early handover intervention is a general human behavior or whether it occurs because the overall process is too slow. It would be interesting to conduct further studies on early handover intervention with different movement speeds to check at what point the movement speed optimally matches the subjects’ grasping.

### 4.3 Limitations

Leichtmann et al. (2022) show in their article that HRI user studies face similar problems of replicability as other behavioral science disciplines. Accordingly, this section highlights the main limitations of this study.

The sample of the study came from the KIT environment. It is quite conceivable that the investigated sample has a higher affinity for technology and thus fewer inhibitions to interact with a robot than the basic population of all people. In this study, a commercially available Franka Panda robot including a gripper was used. The capacitive proximity sensor technology used to trigger the handover of the object and to record the physical handover time is a prototype development that can be requested from the IPR for replication or further development of the experimental setup. Further, regarding the hardware used, it should be noted that capacitive proximity sensing works best with conductive materials and materials with high electrical permittivity. Accordingly, this type of sensing is not equally applicable to every type of object property. The threshold value for the capacitive proximity sensors to release the object was pre-set to a fixed value. However, the threshold value depends on several factors. The area of the object being grasped, and therefore the size of the subject’s hand, as well as skin conductance and ambient humidity can all affect how well the sensor works. For future research, a calibration of the sensor to the respective subject could be developed. However, calibration will also lead to initial habituation. With prior knowledge and habituation, it is not possible to investigate how subjects intuitively adapt to the robot.

The speed of movement of the subjects was not recorded in this study. We focused on conducting the study using the robot’s integrated sensor technology. The detailed recording of the subjects’ movement speed as well as their acceleration and deceleration behavior could provide important additional information for the evaluation and interpretation of the subjects’ behavior. The measurement parameters of physical handover time and early handover intervention have the potential to be collected as standardized metrics across a variety of different measurement systems, such as marker-based motion capture systems or capacitive proximity sensors. As requested by Steinfeld et al. (2006) and Aly et al. (2017), the two measurement parameters should contribute to an easier and fairer comparison between different technical solutions. In this context, appropriate comparative studies of the respective measurement systems need to be carried out.

In the presented study, the robot arm was statically mounted on the table where the handovers took place. The subjects were fully focused on receiving the object from the robot. The transferability of the results of this study should be tested in a more practical context. The type of robotic setup used is well conceivable in an assembly setting, but the focus will be more on the assembly task, which is why further studies should be conducted in the future on handovers without focusing on the handover object while performing a secondary task. In the context of social care robots, it is important to consider that the physical handover of the object is preceded by an approach to the person by a mobile platform of the robot. Therefore, it should be investigated in the future whether the results of this study are also valid when the physical handover is preceded by an approach of the robot to the person.

## 5 Implications

In the course of this study, it has been shown that the collected data can change significantly during the runs. These changes in the data can be attributed to the adaptation of the subjects to the robot and to the handover task. Therefore, in human-robot interaction studies, it is recommended to always have a sufficient number of repetitions of a movement and to perform at least two, preferably three or more runs per test condition.

Based on this study, it is recommended to use comparable metrics when designing human-robot or robot-human handover studies to make studies with similar focus comparable. The physical handover time and early handover intervention metrics have the potential to be collected using a variety of different measurement systems, such as marker-based motion capture (like VICON, ARTTRACK, *etc.*) or capacitive and other sensors integrated into the robot. The measurement parameters can be easily integrated into robotic systems with appropriate computational routines and provide a great scientific added value in human-robot studies.

It is also important to understand the context of use and the user’s goals for using a robot with the appropriate capabilities. If a person actively wishes to have an object handed to them, the final impulse should come from the receiver. If the attention of the receiver is focused on the object to be handed, the handover should be designed so that the user actively grasps the object rather than transporting it completely to the hand. On the other hand, if the goal is to blindly grasp the object without the receiver’s visual attention, this requires adaptation to the receiver’s hand. If the attention of the receiver is not focused on the object being handed, then the object should be transported to the hand so that physical contact is made and the receiver can react to it.

The study showed that an adaptive transport method does not lead to faster physical handovers than a non-adaptive transport method. As the technical development in the field of robot-human or human-robot handovers progresses, it becomes more important to pay more attention to the research field of human motor control and especially to the research in the field of motor adaptation. In the past, a lot of research has been conducted and published in the field of motor adaptation (Krakauer et al., 2019). The theories behind motor adaptation processes need to be applied to joint actions between humans and human-robot interactions. It is important to use this knowledge to tailor robot adaptation mechanisms to the humans who will later use the robot. In conclusion, this study has provided valuable insights into the factors that influence the success of object handovers between robots and humans. Overall, this study makes a significant contribution to the field of human-robot interaction by providing a deeper understanding of how to design effective object handovers.

## Data Availability

The raw data supporting the conclusion of this article will be made available by the authors, without undue reservation.
